# Temperature Grid Sensor for the Measurement of Spatial Temperature Distributions at Object Surfaces

**DOI:** 10.3390/s130201593

**Published:** 2013-01-25

**Authors:** Thomas Schäfer, Markus Schubert, Uwe Hampel

**Affiliations:** 1 Helmholtz-Zentrum Dresden-Rossendorf, Institute of Fluid Dynamics, P.O. Box 510119, 01314 Dresden, Germany; E-Mails: m.schubert@hzdr.de (M.S.); u.hampel@hzdr.de (U.H.); 2 Technical University Dresden, AREVA Endowed Chair of Imaging Techniques in Energy and Process Engineering, 01062 Dresden, Germany

**Keywords:** temperature grid sensor, temperature measurement, wire-mesh sensor, thermal surface monitoring

## Abstract

This paper presents results of the development and application of a new temperature grid sensor based on the wire-mesh sensor principle. The grid sensor consists of a matrix of 256 Pt1000 platinum chip resistors and an associated electronics that measures the grid resistances with a multiplexing scheme at high speed. The individual sensor elements can be spatially distributed on an object surface and measure transient temperature distributions in real time. The advantage compared with other temperature field measurement approaches such as infrared cameras is that the object under investigation can be thermally insulated and the radiation properties of the surface do not affect the measurement accuracy. The sensor principle is therefore suited for various industrial monitoring applications. Its applicability for surface temperature monitoring has been demonstrated through heating and mixing experiments in a vessel.

## 1. Introduction

Temperature field measurements are common and required in many industrial processes as well as in many inspection, analysis and surveillance tasks. Examples are the monitoring of temperature distributions in and around plant components in the processing industry, mineral oil processing equipment or in power generation but also for instance the detection of heat losses in buildings. Considering industrial processes, temperatures are commonly measured locally at selected positions within apparatuses, vessels or pipes or at the surface of components. However, monitoring of temperature fields is beneficial to identify critical surface temperatures, to detect thermal shocks and temperature endurance of plant components, and thus, to prevent early safety shutdowns [1–4]. Furthermore, measuring surface temperature fields allows derivation or even reconstruction of inner surface temperature history or internal flow conditions [5,6] and gives indications of process-related temporal material stress or the development of process runaway conditions [7–9].

Today there are two common ways to measure and visualize temperature fields. Either the surface of an object of interest is equipped with a set of temperature sensors (e.g., thermocouples or thermal resistors) and the temperatures are measured directly, or an infrared camera is used to record images of the emitted thermal radiation. Both approaches have considerable drawbacks. A multi-sensor arrangement requires many electronic processing channels and a lot of cabling. Infrared cameras, however, require optical access to the emission surface, which is not always possible, for instance when a thermally isolated component surface is to be inspected or monitored. Another approach for surface temperature field measurement is the use of temperature sensitive materials, which change their optical properties. Thus, surfaces can be coated by thermochromic liquid crystals, heat sensitive crystalline solids and paints or thermographic phosphor and changes in optical properties can be observed remotely. Such methods are considered as semi-invasive. A detailed review on various measurement techniques can be found in [10]. In this paper a new sensor for the measurement of component surface temperature fields is introduced. The sensor consists of multiple electrical resistance elements assembled in a grid-like structure [11]. The acquisition of the temperature-dependent resistance data of the sensor elements is realized with a special multiplexing scheme, which was adapted from the conductivity wire mesh sensor [12] frequently used for gas-liquid flow measurements. While the latter measures the conductivity distribution in the crossings of a wire grid exposed to a multiphase flow, the temperature grid sensor now uses passive conversion elements (thermal resistors) to produce a map of temperatures from multiplexed conductivity readings. As a proof of principle, a sensor was built and applied to measure transient temperature fields on the wall of a vessel in heating and mixing scenarios.

## 2. Electrical Sensor Design and Measuring Electronics

**Figure 1.**
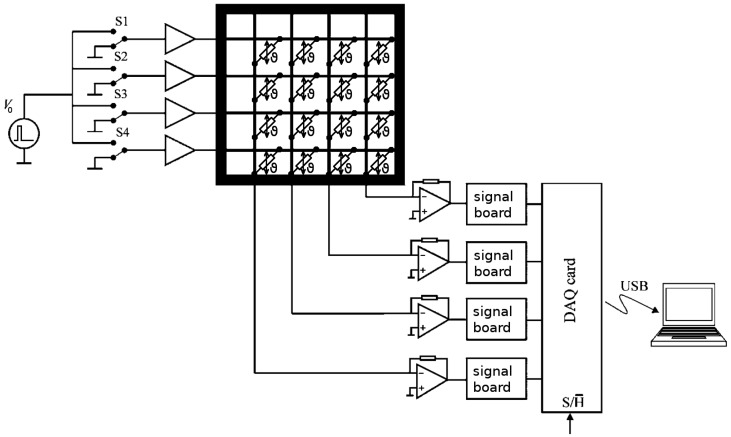
Simplified scheme of the temperature field sensor and its electronics.

Figure 1 shows the simplified scheme of the sensor and the electronics. The sensor is a regular grid of wire electrodes with platinum resistors in the crossings. The transmitter electrodes (horizontal wires in Figure 1 are activated consecutively by a multiplexer. That is, a voltage is applied to the active wire while the other ones are kept grounded. Electrical currents flowing from the active transmitter wire through the resistors to all receiver electrodes (vertical wires in Figure 1 are measured in parallel. This scheme allows rapid temperature measurements with sampling frequencies up to 1 kHz while the cabling requirements and number of electronic channels are low.

**Figure 2.**
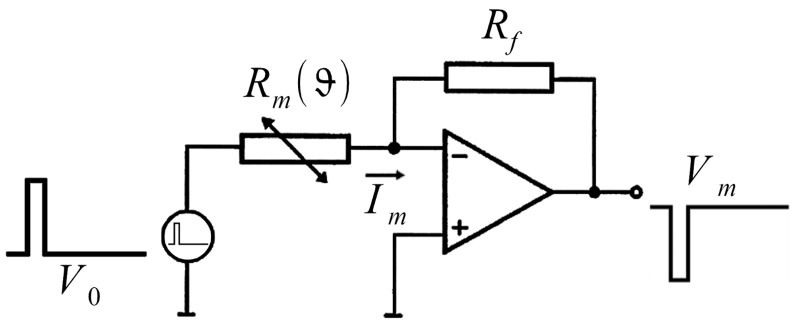
Equivalent circuit for one electrical resistance element.

Figure 2 shows an equivalent circuit for one crossing point with the temperature dependent electrical resistance element. The pulsed excitation voltage *V*_0_ causes an electrical current *I_m_* in the temperature dependent electrical resistance element *R_m_*(*ϑ*). The current *I_m_* is transformed into a voltage *V_m_* using a transimpedance amplifier. Assuming that the operational amplifier is ideal, the measured voltage is determined by equation Equation (1) with the feedback resistance *R_f_*, the thermal resistor value *R_m_*(*ϑ*) and the excitation voltage *V*_0_.

(1) $$V_{m}=-V_{0}\cdot \frac{R_{f}}{R_{m}(\vartheta )}$$

The voltage signals of all receiver channels are further amplified, digitized and stored in a memory of a special data logger. For the prototype temperature field sensor Pt1000 platinum chip resistors (Jumo type PCA 1.2005.10 E55) were used. Due to the thermal inertia of the temperature sensors (response time *t*_0.9_ = 0.3 s in water) and to satisfy the Nyquist sampling criterion, a minimum sampling frequency of 6.6 Hz is necessary. To allow noise reduction by oversampling and digital filtering, a sampling frequency of 100 Hz has been chosen with excitation pulses of 12 μs length. Since the resulting currents through the resistors are less than 1.0 mA, drift effects due to self-heating of the platinum chip resistor can be neglected. For the Pt1000 element the effect of the temperature on the resistance is given by equation Equation (2).

(2) $$R_{m}(\vartheta )=R_{0,Pt}\cdot (1+\alpha_{Pt}\cdot \vartheta )\;\text{for}\;0{}^{\circ}C\leq \vartheta <220{}^{\circ}C$$

*R*_0,*Pt*_ is the nominal resistance and *α_Pt_* is a specific temperature coefficient of the platinum resistor. If *R_f_*, *R*_0,*Pt*_ and *α_Pt_* are known, temperature can directly be determined from the measured voltage according to equation Equation (3).

(3) $$\vartheta =\frac{C_{1}}{V_{m}}+C_{2}\;\text{with}\;C_{1}=-\frac{V_{0}\cdot R_{f}}{R_{0,Pt}\cdot \alpha_{Pt}},C_{2}=-\frac{1}{\alpha_{pt}}$$

Alternatively, the constants *C*_1_ and *C*_2_ can be determined from calibration measurements at different temperatures. This also takes into account the effects of the different length of path of the connection cables in the matrix.

## 3. Experimental Set-up and Prototype Sensor

**Figure 3.**
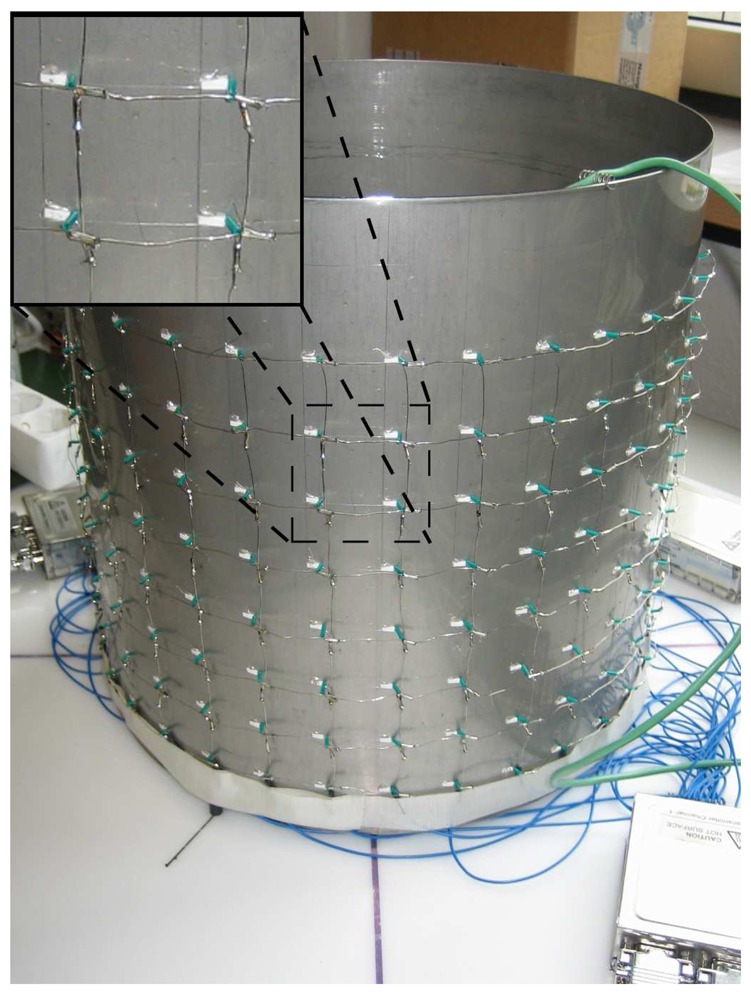
Mixing vessel with mounted temperature field sensor and zoomed view into a 2 × 2 sensor element matrix.

As a proof of principle, a prototype temperature grid sensor was built and used in heating and mixing experiments in a metallic vessel. The stainless steel mixing vessel (Figure 3) has an inner diameter of 300 mm. A matrix of 8 × 32 temperature sensitive platinum chip resistors was mounted at the wall of the vessel using a special thermal adhesive (Arctic Silver Thermal Adhesive). The locations of the sensor elements as well as vessel dimensions are summarized in Table 1. To reduce thermal influences of the environment, such as heat dissipation, the vessel was thermally insulated (not shown in Figure 3).

For the calibration measurements, the vessel was filled with water at two different temperatures and the constants *C*_1_ and *C*_2_ (Equation (3)) were derived for all matrix points. For each calculation, the mean values of 3,000 calibration measurement values were used. An additional lance with eight thermocouples (mantle thermocouple, type K NiCr/NiAl, 1 mm) was installed inside the vessel close to the wall to measure internal temperature profiles for comparison with the wall temperatures field.

## 4. Heating and Mixing Scenarios

### 4.1. Heating Experiment

**Figure 4.**
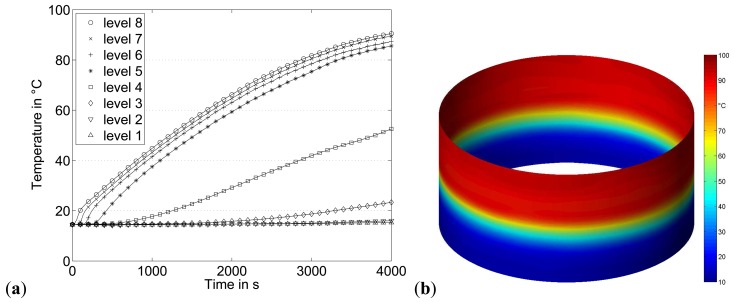
(**a**) Temperature evolution at different vessel heights during the heating procedure, (**b**) vessel wall temperature field at t = 4,000 s.

For the purpose of heating the liquid in the vessel (non-agitated), a heating element (Weltor 1,000 W) was installed approximately 100 mm above the vessel bottom in the centre. The temperature evolution resulting from the heating process at different sensor heights is shown in Figure 4(a). Here, temperatures of sensor elements located at the same level were averaged. The temperatures at the four upper levels show a similar behaviour, while the temperature increase at level 3 and level 4 (zone of the heating element) is delayed.

Figure 4(b) shows the temperature field at the wall of the vessel 4,000 s after start of the heating. The higher spatial resolution of the shown temperature distribution was generated by bicubic interpolation based on the measurement data. The development of two thermal layers separated by a thin transition layer can be clearly observed. The heating element induces a liquid circulation in the heating zone above the heating element. However, the liquid fraction below remains stable at lower temperature.

### 4.2. Mixing Experiment

**Figure 5.**
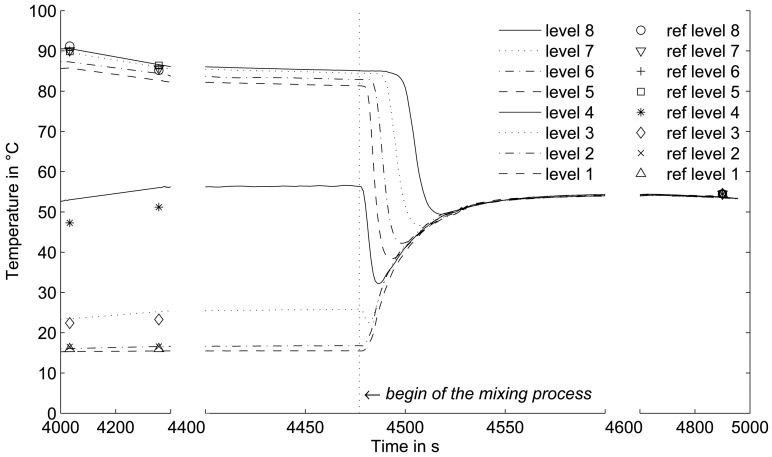
Temperature evolution during mixing of the stratified liquid.

For an assessment of the sensor dynamics, a mixing experiment was conducted in the temperature-stratified liquid-filled vessel (see Figure 4(b)). Therefore, a stirrer (type: MLW MR25 with impeller mixer, typical flow: radial) was installed in the centre of the vessel with blades 20 mm above the vessel bottom. For that reason, the heater was installed slightly off the centre. The stirrer was operated at a stirrer speed of 50 rpm and the mixing process was started at the time that is indicated by the dashed line in Figure 5. The temperature evolution at all levels was measured until uniform temperature conditions were achieved. Again, the temperatures of sensor elements located at the same height were averaged. While temperatures at the lower levels 1 and 2 show a rapid and continuous increase, the upper zones display a sudden decrease of the temperature even below the later achieved equilibrium temperature. Here, the stirrer together with the heater as a vortex breaker causes an axial liquid circulation with cold liquid from the bottom region flowing upward near the wall while the heated water flows downward in the vessel centre. Already after a short recirculation time temperature homogenizes in the vessel.

**Figure 6.**
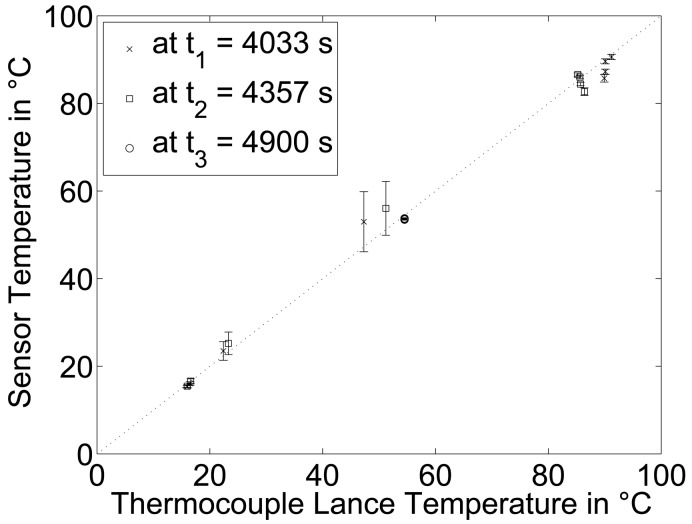
Mean values and standard deviations of sensor data versus thermocouple lance temperature readings during the mixing procedure.

Furthermore, reference temperature data were taken prior to the mixing and after uniform temperature was achieved. The reference temperatures were measured with the thermocouple lance at the same heights as the temperature grid sensor measurement planes and they are indicated by the symbols in Figure 5. In the parity plot in Figure 6, the temperatures measured with the thermocouple lance and from all 256 grid sensor elements are compared. While good agreement was found for level 1 to 3 and 5 to 8, clear deviations prior to the mixing were measured at level 4, which represents the transition zone characterized by strong temperature gradients.

### 4.3. Experiment with a Moving Heat Source

**Figure 7.**
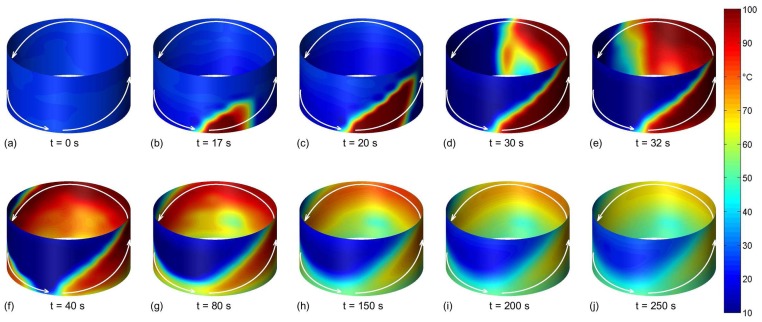
Temperature fields during the moving heat source experiment.

To show the ability to measure transient spatial temperature distributions, a moving heat source was applied. A hot liquid jet (96 °C) with constant flow rate was injected in the empty vessel from a pipe moving 360° along the wall, thereby drawing an ellipse within 40 s (Figure 7(a–f)) as highlighted by the arrows in Figure 7. Here, the liquid jet forms a film flowing downward on the wall.

Figure 7 shows the temporal evolution of the temperature field at the surface of the vessel wall. As in Figure 4(b), the higher spatial resolution of the shown temperature distributions was generated by bicubic interpolation based on the measurement data. The elliptic path of the jet can be clearly visualized by the sensor. The images also show that the downward flowing liquid film heats the lower wall regions. The further evolution of the surface temperature field after the injection has been stopped is shown in Figure 7(g–j). Here, the temperature field changes due to heating by remaining liquid films, heat conduction in the wall and heat losses.

### 4.4. Temporal Sensor Response

**Figure 8.**
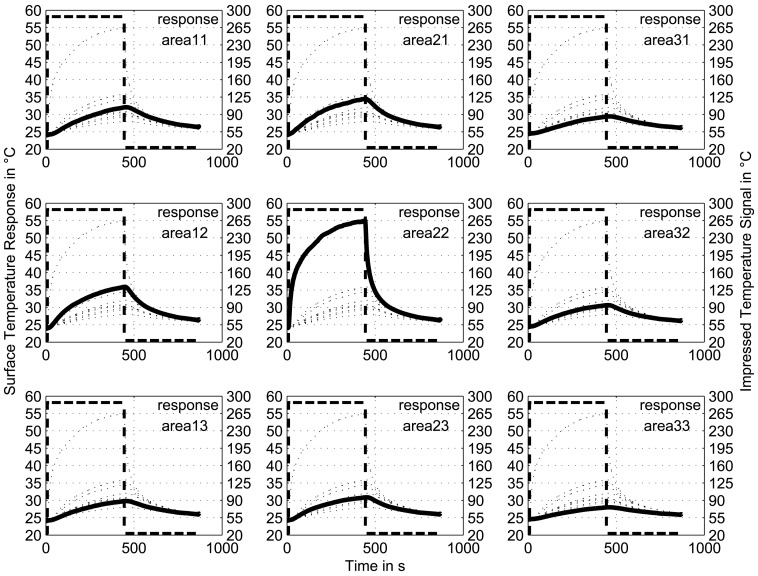
Sensor response. The dashed curve represents the impressed temperature signal, the bold curve indicates the response of the particular area and the dotted curves show the responses of the other eight areas. As an illustration, the positions of the nine diagrams are similar to the location of the nine sensor areas.

To characterize the response of the installed temperature grid sensor, a temperature step was locally impressed inside the vessel in the area of one sensor element at level 5 (see “area 22” in the embedded scheme in Figure 8). The response of the installed temperature sensor was analysed for a 3 × 3 sensor element matrix neighbouring the heated element (see “area 11” to “area 33”).

Figure 8 shows the temperature step and the corresponding temperature signals. The responses of the sensor elements are clearly lower in amplitude than the impressed temperature step, which can be attributed to the heat removal from the vessel wall due to the large surface and high heat capacity. Nevertheless, the shapes of the curves represent the response characteristics of this temperature sensor configuration. The highest response was observed for the centre element of the matrix. The delayed temperature increase of the surrounding elements occurs due to the heat conductivity within the metallic wall. Additionally, the heat transfer caused by free convection near the wall inside the vessel plays a role. However, the temperature increase is lower. In summary, the influence of the wall material heat conductivity on the neighbouring areas is not negligible, but the sensor still resolves spatial variations in local temperature precisely.

## 5. Conclusions

In this paper a new wire-mesh based sensor for the measurement and visualization of surface temperature fields was introduced. Multiple platinum chip resistors (Pt1000) are assembled in a matrix structure and can be read-out with high frequency using a multiplexing-driving scheme. The sensor can be installed at unevenly shaped and insulated object surfaces, where for instance no optical access for infrared camera observation is possible. The applicability of such a sensor concept, e.g., for monitoring critical surface temperatures, occurrence of thermal shocks and temperature endurance of plant components, was demonstrated by some basic heating and mixing experiments in a large vessel.

## Figures and Tables

**Table 1.** Dimensions and specifications of vessel and sensor.

**vessel**	
height	300 mm
inner diameter	300 mm
wall thickness	1 mm
material	stainless steel
**measurement points**	

number in radial direction	32
number in axial direction	8
spacing in radial direction	30 mm
spacing in axial direction	30 mm
**measurement planes** (height above bottom)	

level 1	30 mm
level 2	60 mm
level 3	90 mm
level 4	120 mm
level 5	150 mm
level 6	180 mm
level 7	210 mm
level 8	240 mm
